# NLRP3 inflammasome: The rising star in cardiovascular diseases

**DOI:** 10.3389/fcvm.2022.927061

**Published:** 2022-09-20

**Authors:** Yidan Zheng, Li Xu, Nianguo Dong, Fei Li

**Affiliations:** Department of Cardiovascular Surgery, Union Hospital, Tongji Medical College, Huazhong University of Science and Technology, Wuhan, China

**Keywords:** cardiovascular diseases, NLRP3 inflammasome, interleukins, inflammation, pyroptosis

## Abstract

Cardiovascular diseases (CVDs) are the prevalent cause of mortality around the world. Activation of inflammasome contributes to the pathological progression of cardiovascular diseases, including atherosclerosis, abdominal aortic aneurysm, myocardial infarction, dilated cardiomyopathy, diabetic cardiomyopathy, heart failure, and calcific aortic valve disease. The nucleotide oligomerization domain-, leucine-rich repeat-, and pyrin domain-containing protein 3 (NLRP3) inflammasome plays a critical role in the innate immune response, requiring priming and activation signals to provoke the inflammation. Evidence shows that NLRP3 inflammasome not only boosts the cleavage and release of IL-1 family cytokines, but also leads to a distinct cell programmed death: pyroptosis. The significance of NLRP3 inflammasome in the CVDs-related inflammation has been extensively explored. In this review, we summarized current understandings of the function of NLRP3 inflammasome in CVDs and discussed possible therapeutic options targeting the NLRP3 inflammasome.

## Introduction

Cardiovascular diseases (CVDs) pose a major risk to public health in modern society. The percentage of U.S. adults with a 10-year predicted atherosclerotic CVD risk of ≥20% has reached 13.0% from 1999 to 2000. And between 1990 and 2010, the global number of deaths per year attributed to cardiomyopathy and myocarditis increased by 40.8%, from 286,800 to 403,900 (1). Since Rokitansky and Virchow pointed out the role of immunology in atherosclerosis progression in the 19th century (2), a growing number of CVDs have been found to be tightly associated with inflammation. Although lots of evidence has shown that inflammatory processes have great importance in the development of CVDs, the specific mechanism is still not completely clear.

Nucleotide oligomerization domain-, leucine-rich repeat-, and pyrin domain-containing protein 3 (NLRP3) is a pattern recognition receptor (PRR). It belongs to the NOD-like receptor (NLR) family and is a crucial sensor of cardiovascular tissue damage. NLRP3 can be activated by various kinds of diverse immune stimuli and promotes the maturation of IL-1β and IL-18 in IL-1 family cytokines. IL-1β and IL-18 involve both innate and adaptive immune responses, performing a significant role in inflammatory development of atherosclerosis, cardiomyopathy, abdominal aortic aneurysm, calcific aortic valve disease, heart failure, and cardiovascular and cerebrovascular ischemic injury. In this review, we summarized recent advances focusing on the role of NLRP3 inflammasome in atherosclerosis, cardiomyopathy, and other common CVDs, and we discussed the therapeutic potential of inhibiting NLRP3 inflammasome.

## NLRP3 inflammasome

Since first reported in 2002 (3), the inflammasome has become the predominant research subject in innate immunity. NLRP3 expression has been observed not only in leucocytes but also in cells of the cardiovascular system such as vascular endothelial cells, vascular smooth muscle cells and cardiomyocytes. Intracellular PRRs play a critical role by directly inducing the formation of inflammasome. Among them, five intracellular PRRs are recognized as inflammasome receptors: pyrin, NLRP1, NLRP3, NOD-, LRR-, caspase recruitment domain (CARD)-containing protein 4 (NLRC4), and absent in melanoma 2 (AIM2) (4). Several researches have revealed that pyrin, NLRP1, NLRP3, NLRC4, and AIM2 have their specific functions and triggers (5). Distinguished from them, NLRP3 is unique since it can be activated by a variety of pathogen-associated molecular patterns (PAMPs) and damage associated molecular patterns (DAMPs) (5).

The NLRP3 inflammasome is composed of three parts: NLRP3, apoptosis-associated speck-like protein containing a CARD (ASC), and pro-caspase-1 (6).

The NLR proteins share a mutual organization: the central domain NOD (also known as NAIP, CIITA, HET-E, and TP-2 [NACHT]) (7). the C-terminal LRR domain, and the N-terminal effector domain (8, 9). NLRP3 has the common structure of NLR, with a central NOD domain and the LRR, and employs pyrin as the N-terminal effector.

ASC is a speck-like protein, serves as the connection between NLRP3 and pro-caspase-1. It consists of two domains: a C-terminal CARD and an N-terminal PYD (10). CARD on ASC can be connected with pro- caspase-1 *via* the homotypic CARD–CARD reaction, and PYD on ASC provides a tight connection with pyrin on NLRP3 through a similar interaction (11–13). Besides recruiting pro-caspase-1 and connecting with PYD on NLRP3, the oligomerization of pro-caspase-1 on ASC filaments enables proximity-driven autocatalytic caspase-1 maturation (14).

Pro-caspase-1 is the effector protein of the inflammasome. After autocatalytic maturation, pro-caspase-1 is cleaved into caspase-1. Cleaved caspase-1 is a vital proteolytic enzyme in human homeostasis. Previous research has already found that caspase-1 tended to form an active heterotetramer, cleaving pro-IL-1β and pro-IL-18 into mature cytokines and inducing their release (15).

Inflammasome assembly is organized subsequently in macrophages. Toll-like receptors (TLRs) are widely expressed on the membrane of innate immune cells. Activation of TLRs by PAMPs or DAMPs leads to the phosphorylation and lysis of inhibitor of NF-κB (IκB) and releases the active NF-κB. Then NF-κB enters the nucleus and primes the transcription of pro-IL-1β, pro-IL-18, and NLRP3 (16). Besides the canonical TLR–NF-κB pathway, IL-1β receptor (IL-1R) and tumor necrosis factor receptor (TNFR) can perform similar functions in the priming of NLRP3. IL-1R and TNFR can activate the NF-κB pathway and then lead to the transcription of NLRP3, which indicates that IL-1β, the product of the NLRP3 inflammasome, can also promote the priming of NLRP3 itself (17).

Besides the well recognized common regulation, recent findings demonstrated circadian oscillations in NLRP3 expression process. It was first observed in mouse and human primary macrophages (18), and multiple nuclear receptors, besides the classic NF-κB, participate in this regulation of rhythmic pattern. Retinoic acid receptor-related orphan receptor γ (RORγ) and Rev-erbα belong to nuclear receptors family. *In vivo* and *in vitro* experiments reported that these two receptors directly bind to the same site in the promoter and thereby control the priming of NLRP3 (18, 19). Additionally, Rev-erbα is also circumstantially involved the NF-κB signaling pathway and negatively regulate the priming and activation of NLRP3 inflammasome (20, 21). Although the perspective of time-dependent regulation of NLRP3 expression is novel, it has been accepted and will facilitate the further exploration and reasonable explanation of biorhythms in inflammation and cardiovascular diseases development.

One of the direct results of NLRP3 activation is pyroptosis. Pyroptosis is a distinct programmed cell death, characterized by pore formation in the plasma membrane, cell swelling, cell osmotic lysis, and release of pro-inflammatory intracellular contents (22, 23). In pyroptosis, inflammasome serves as a mediator. The detailed mechanism was described as below: caspase-1 on NLRP3 inflammasome and splits a unique protein, gasdermin D (GSDMD), into two fragments; the N-terminal fragment ruptures the membrane to form pores, releasing IL-1 and IL-18 and leading to the pyroptosis process (24).

## Activation of the NLRP3 inflammasome

**Figure 1:**
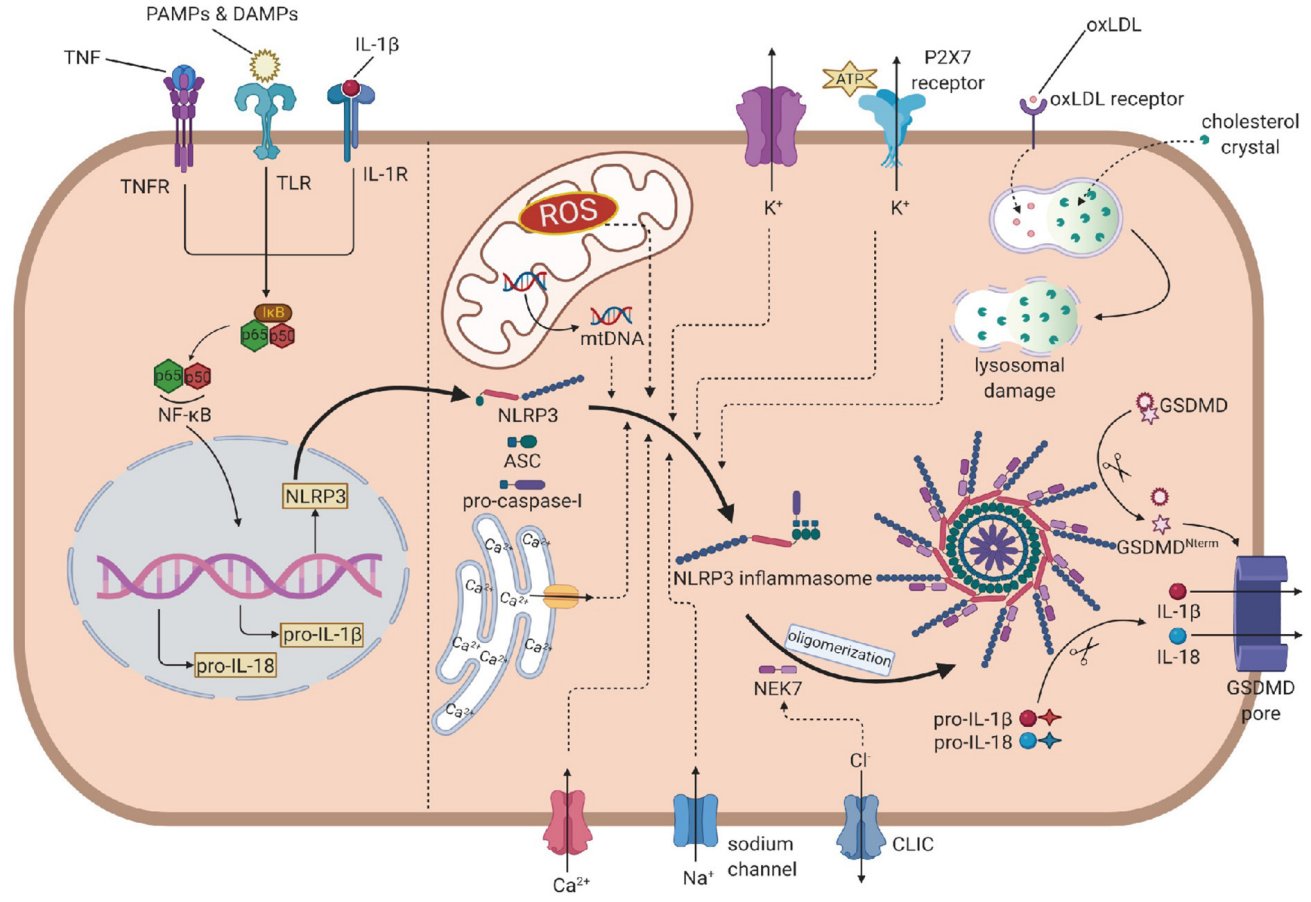
The overall mechanism of the NLRP3 inflammasome activation. In the priming step, PAMPs, DAMPs, IL-1β and TNF induce the phosphorylation and lysis of inhibitor of NF-κB (IκB), activate the NF-κB pathway, leading to the transcription of NLRP3, pro-IL-1β and pro-IL-18. In the activation process, NLRP3 recruits ASC, which links to pro-caspase-1, to assembly the inflammasome. Then, NEK7 is attached to inflammasome and induces the oligomerization. The efflux of K+, influx of Na+ and Ca2+ (from extrecellular and endoplasmic reticulum), ROS, mtDNA and oxLDL or cholesterol crystal- induced lysosomal damage contribute to the assembly. The efflux of Cl- promotes the attachment of NEK7 and then the oligomerization. Caspase-1 is activated by autoproteolysis on oligomeric NLRP3 inflammasome, cleaving pro-IL-1 and pro-IL-18 into IL-1 and IL-18. Caspase-1 also cleaves GSDMD to form the GSDMD pore, leading to the release of IL-1 and pyroptosis. NLRP3, nucleotide oligomerization domain (NOD)-, leucine-rich repeat (LRR)- and pyrin domain (PYD)-containing protein3; PAMP, pathogen- associated molecular patterns; DAMP, damage associated molecular patterns; TNF, tumor necrosis factor; NF-κB, nuclear factor κ-light-chain enhancer of activated B cells; ASC, apotosis-associated speck-like protein containing a CARD; NEK7, NIMA-related kinase 7; NIMA, never in mitosis gene A; ROS, reactive oxygen species; oxLDL, oxidized low-density lipoprotein; GSDMD, gasdermin D.

The activation of NLRP3 inflammasome is strictly regulated at various levels, requiring two independent stimuli. The first signals are mainly PAMPs and DAMPs, activating the PRRs or other receptors and inducing the transcription of NLRP3, pro-IL-1β, and pro-IL-18 *via* the NF-κB pathway. The second signal activates NLRP3 and promotes the assembly of its inflammasome (16). Generally, stimulus events are divided into three main categories: ionic flux, reactive oxygen species (ROS) (25), and damage or dysfunction of cell organelles (Figure 1).

### Ionic flux

Before the discovery of inflammasome, cytosolic depletion of K+ has been proved to promote the maturation and release of IL-1β (26). Blocking K+ efflux with high extracellular potassium concentration represented an inhibition of the activation of NLRP3 inflammasome while decreasing intracellular K+ concentration resulted in inflammasome activation. ATP is a common trigger of NLRP3 inflammasome activation, promotes the activation via purinergic receptor (P2X7R) (27). Among these, the engagement of P2X7R is accompanied by K+ efflux. Besides the classic activating pathway, the efflux of K+ would also activate non-canonical pathways, leading to the activation of NLRP3 inflammasome (28, 29).

The mobilization of calcium participates in many intercellular signaling pathways, tightly associated with the activation process. Early studies found that chelating Ca2+ with BAPTA-AM suppressed the secretion of IL-1β, indicating that Ca2+ signaling might involve the NLRP3 inflammasome activation (30). Later reports showed that when knocking out the murine calcium-sensing receptor (CASR), the activation seemed to be restrained (31). However, the specific mechanism is not fully illustrated. Some studies proposed that Ca2+ might contribute to conformational changes, facilitating the interaction between NLRP3 and ASC (31).

Other concentration changes of ions may also play considerable roles in the activation. When blocking Na+ influx by decreasing the extracellular concentration, the threshold of K+ efflux for NLRP3 activation up-leveled, resulting in the blockage of activation. When using monosodium urate crystals to raise intracellular concentrations and block the influx of Na+, the intracellular osmotic pressure rose, leading to water influx and cellular swell, which decreased the relative concentration of potassium, blocking its efflux and the downstream reaction: activation of the NLRP3 inflammasome (32). Chloride ion is also involved in the activation as increasing extracellular chloride concentrations promotes the maturation of IL-1β induced by ATP (33). Recent studies provided some new perspectives of explanations: on the one hand, chloride intracellular channels (CLICs) can promote the NLRP3–Nek7 interaction (34), which is a downstream reaction of mitochondrial dysfunction and regulates the activation of the NLRP3 inflammasome. On the other hand, experiments have proved that CLICs involve in the transcription of pro-IL-1β and the formation of ASC–speck, while the NLRP3 level is not infected (35).

### Reactive oxygen species

Reactive oxygen species are a broad category of substances, mainly are the byproducts of various aerobic metabolic processes (36). In the initial study, it was found that ROS shared similar trigger signals to the activation of NLRP3 in the treated cells (36). NADPH oxidase (NOX) is recognized to be the source of ROS. Although generally blocking NOX did not affect the activation of NLRP3 inflammasome in human and mouse cells (37, 38), inhibiting of NOX2 could affect the activation in certain mouse models such as brain injury from ischemic stroke (39). Moreover, NADPH oxidase 4 (NOX4) also participates in the oxidation of fatty acids, which contribute to the activation of NLRP3 (40). In a later study, Bauernfeind et al. provided a new perspective of ROS function, showing that ROS inhibitors also blocked the priming signal of NLRP3 activation (41). From all evidence above, the ROS pathway is highly involved in the NLRP3 activation, but the components are complex and require precise studies to distinguish their functions from each other.

### Mitochondrial dysfunction

The respiratory reaction in mitochondria is a chain reaction. Once the chain process is inhibited, oxygen involved in the respiratory chain could accumulate in the form of mitochondrial ROS (mtROS). Nakahira et al. have already proved that mtROS was required for the response of NLRP3 to LPS and ATP. In sepsis models, mice without mitochondria protective protein exhibited the higher mtROS concentration and greater secretion of IL-1β and IL-18 (42). Another product of mitochondrial dysfunction is mtDNA. In the initial study, it was found that the mtDNA released from dysfunctional mitochondria not only interacted with NLRP3 (43), but also performed oxidization and played a critical role in the activation. Later experiments further evidenced that the synthesis of mtDNA induced by the TLR signal pathway was crucial for the activation process (44).

### Lysosomal damage

Particulate matter has been found to promote the activation of the NLRP3 inflammasome in macrophages (45–47). These particles cannot be decomposed by active lysosomal enzymes, causing the lysosomal disruption and leading to lysosomal acidification and leakage of lysosomal contents into the cytosol. H+ ATPase is a proton pump that maintains the hydrogen equilibrium between lysosomes and the cytosol. After applying Bafilomycin A, the inhibitor of H+ ATPase, particulate matter-induced NLRP3 inflammasome activation was also inhibited, which implied that lysosomal acidification played a remarkable role in the activation process (37). The leakage of active lysosomal enzymes is also reasonable for the particulate matter-induced activation of NLRP3. Cathepsin B is a remarkable lysosomal enzyme. Research pointed out that the release of cathepsin B was required for the release of IL-1β, but did not affect the production of pro-IL-1β. Further research on enzymes like cathepsins L, C, S, and X admitted similar results. Due to the broad substrate specificities of cathepsins, the promoting role of single cathepsin in the activation can be obscured by functional redundancy (48). All in all, leakage of active lysosomal enzymes plays unignorable roles, but their deficiency can also be offset by other pathways.

## The role of the NLRP3 inflammasome in cardiovascular diseases

### Atherosclerosis

**Figure 2:**
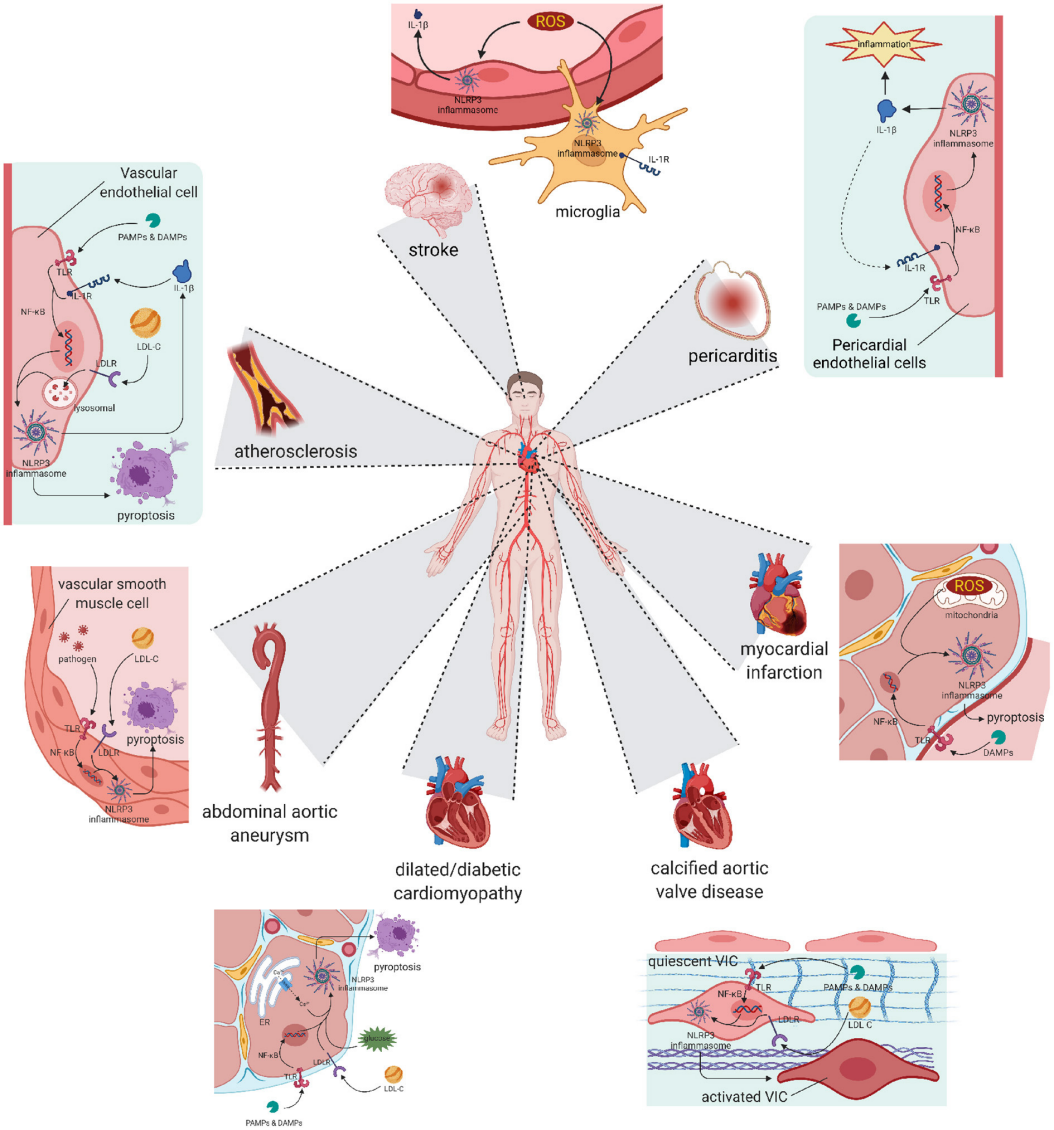
Pyroptosis and differentiation mediated by NLRP3 inflammasome result in various cardiovascular diseases. Atherosclerosis: The activation of NLRP3 happened in vascular endothelial cells leads to cell pyroptosis and subsequent lipid accumulation, which induces the formation of atherosclerotic plaque. Pericarditis: The activation of NLRP3 happened in pericardial endothelial cells can be initiated by pro-inflammatory substances, and also worsens the inflammatory development of pericarditis. Abdominal aortic aneurysm: Activation happened in aorta smooth muscle cells leads to cell loss, resulting in abdominal aortic aneurysm. Myocardial infarction and Stroke: Activation happened in reperfusion cells exacerbates ischemia-reperfusion injury, worsens the myocardial infarction or stroke symptom. Cardiomyopathy: Chronic NLRP3 inflammation triggered by hyperglycaemia or hyperlipidemia promotes pyroptosis of cardiocytes and remodeling of ventricular, ending in dilated or diabetic cardiomyopathy. Calcific aortic valve disease: NLRP3 inflammation in aortic valve can activate quiescent valve interstitial cells into activated valve interstitial cells, initiating the calcific process of valve. NLRP3, nucleotide oligomerization domain (NOD)-, leucine-rich repeat (LRR)-, and pyrin domain (PYD)-containing protein 3.

Atherosclerosis has been widely accepted as the fundamental cause of various CVDs (49) (Figure 2). It is characterized by three main features: passive lipid accumulation, proliferation of vascular smooth muscle cells, and leukocyte infiltration (50). The mechanism was initially attributed to the consequences of lipid deposition (51). However, as research progressed, the involvement of inflammatory reactions was revealed and received much attention.

Many clinical and experimental studies have already implied the involvement of several inflammatory cytokines. In 2010, Duewell et al. were the first to directly reveal the importance of NLRP3 in the process of atherogenesis (45). They used low-density lipoprotein receptor (LDLR)-deficient (LDLR-/-) mice as a model, transplanted bone marrow of wild-type, Nlrp3-/-, Asc-/-, or IL-1α-/-/IL-1β-/- mice, and fed the mice with a western diet (containing 0.15% cholesterol). The results showed that the lesions of mice transplanted with Nlrp3-/-, Asc-/-, or IL-1α-/-/IL-1β-/- marrow were decreased compared to the wild-type ones. In 2012, Usui et al. mated Apoe-/- mice with Casp1-/- mice, producing Apoe-/- Casp-/- mice (50). They fed them with a western diet and found the lesions were also decreased without affecting total serum cholesterol concentrations or the lipoprotein-cholesterol distribution. In the same year, Gage et al. fed Apoe-/- Casp-/- mice with a high-fat diet (containing 1.5% cholesterol) and obtained similar results (52). These results suggested that the inflammatory process caused by NLRP3 was highly involved in the atherogenesis lesion. These results seem not absolute, some studies also obtained contradictory results (53–55). However, those can be explained by differences in gender, feeding conditions and the choice of experiment models. A LDLr-/- mouse model may be more preferred for mimicking human atherosclerotic pathology.

The detailed mechanism varies in different studies, but cholesterol crystals are commonly considered as the first and central trigger of the activation of the NLRP3 inflammasome (56, 57). Similar to the other crystalline NLRP3 activators, CCs activate NLRP3 via lysosomal damage (58). Previous experiments have proved that upregulating the expression of transcription factor EB, which activates lysosomal and autophagy genes in response to lysosomal stress, resulted in the reduction of lysosomal dysfunction and secretion of IL-1β (59). Similarly, Apoe-/- mice with macrophage-specific autophagy deficiency treated with increased CCs developed enlarged atherosclerotic plaques (60). These results also signify the atheroprotective function of macrophage autophagy in the decrease of CCs-mediated NLRP3 activation.

Besides the classic lysosomal damage activation pathway, CCs also contribute to the priming of the NLRP3 activation process. In whole blood composition analysis, CCs were shown to activate the complement system (61), which provides the priming signals for activation *in vivo*. Additionally, CCs also induce the release of neutrophil extracellular traps (NETs), which were proved to involve in the priming process (62). Experiments with NET-deficient mice showed that NET-related enzymes such as neutrophil elastase (NE) and neutrophil proteinase 3 (PR3) were not detected, which was later reported to be involved in the cleavage of pro-IL-1β. Naturally, decreased atherogenic lesions were also observed in the NET-deficient mice (63).

LDL-cholesterol (LDL-C) is another significant substance in the activation of NLRP3 (64). Since the blood cholesterol levels (mainly LDL-C) were found to be tightly correlated with the occurrence of atherogenesis, LDL-C was naturally considered as a notable risk factor for CVDs (65). Classic conservative therapy focuses on controlling plasma cholesterol levels and has been recognized to receive effective result in reducing cardiovascular event risk. However, this treatment is not sufficient for all patients (66) and patients with clonal hematopoiesis may not receive ideal alleviation from to statin or ezetimibe treatments. LDL is consumed by the body through enzymatic or non- enzymatic mechanisms to create oxLDL, which is the real culprit of atherogenesis (67, 68). Cluster of differentiation (CD) 36 is a kind of scavenger receptor (SR) that can mediate the internalization of oxLDL into macrophages (69). After being taken up, oxLDL induces the formation of intracellular crystalline cholesterol, which activates the assembly of the NLRP3 inflammasome via lysosomal damage as mentioned above (45). In addition to the activation, recent research also discovered that oxLDL could bind to CD36 and promoted the assembly of TLR4/6 heterodimers, leading to the priming of the activation (70).

Other than these two major pro-atherosclerotic substances, some small molecules such as calcium phosphate crystals and ATP are tightly associated with vascular calcification and calcified atherosclerotic lesions. Calcium phosphate crystals involve in the NLRP3 activation process similar to CCs, affect the caspase-1 pathway, and then promote the cleavage and release of IL-1α and IL-1β (50). Moreover, extracellular ATP released by dead cells is also a significant trigger of activation, while P2X7R is a central receptor mediating the ATP-dependent activation pathway. It was found that the expression of P2X7R increased in LDL-/- and Apoe-/- mice. Accordingly, when P2X7R was knocked down in LDL-/- mice, the lesions were observed to be limited. Similar results were obtained in Apoe-/- and P2X7R-deficient mice. These indicate that extracellular ATP activates the ATP-dependent NRLP3 inflammasome activation pathway, and therefore induces atherogenesis (71, 72).

One study in 2018, however, found that IL-1β treatment failed to limit the size of lesion size, but inhibited the beneficial outward remodeling. Smooth muscle cells and fibrous caps were found to be deficient in SMC-specific knockout of IL-1r1 mice after 18 and 26 weeks of Western diet feeding (73). These results indicated that it is not appropriate to simply summarize the role of IL-1β as a negative effect in the atherosclerotic development. Nonetheless, IL-1β also promotes vascular calcification, plaque rupture and vascular wall stiffness, the positive effect of IL-1β is still controversial.

### Pericarditis

Pericarditis is a common disorder in pericardium with a vast array of infectious or non-infectious causes (74). Although generally benign and self-limiting, the risk of various complications and recurrences reached over 30% in affected population under the standard therapies (75). However, the pathophysiology of pericarditis has remained poorly demonstrated, and therefore only limited drugs was approved for the treatment of acute pericarditis (76).

Colchicine is now considered first-line therapy for both acute and recurrence pericarditis (77). It is known as an inhibitor of microtubule polymerization for anti-inflammatory therapies. Recent findings demonstrated that colchicine inhibits the activation of NLRP3 inflammasome with the capability of blocking microtubule assembly and impairing irritant agent presentation (78, 79). Moreover, the outcome of Anakinra—Treatment of Recurrent Idiopathic Pericarditis (AIRTRIP) study showed that anakinra, a recombinant IL-1 Ra, effectively reduce the risk of recurrence in pericarditis patients with colchicine- resistant (80). These clues hint the NLRP3 inflammasome/IL-1 signal pathway may play a central role in the process of pericarditis.

Prior to the *in vivo* experiments, the examination was conducted on pericardial samples from patients with chronic pericarditis to explore the level difference in inflammatory molecules (81). Immunofluorescence staining result showed that ASC increased in human pericardial samples compared to the control ones. The expression of NLRP3 and caspase-1 were also observed significantly upregulated in pericarditis cases vs. controls, which echoed the hypothesis from pharmaceutical treatments, emphasizing the essence of the development in pericarditis (81).

Mauro et al. were the first to confirm the treatment effect of inhibiting NLRP3 inflammasome in acute pericarditis mouse model (82). Zymosan A, a yeast-derived activator of the NLRP3, was injected in the pericardial sac of CD-1 male mice to induce the pericarditis model. Three parameters, presence of pericardial effusion, pericardial thickness, and ASC expression, were measured to evaluate the inflammation of the pericardium. Three days after surgery, a larger effusion was observed in treated mice compared to sham mice. Seven days after surgery, expression of ASC and visceral pericardial thickness posed a consistency of increasing (81). In following experiments of recovery, blocking NLRP3 inflammasome with nonsteroidal anti-inflammatory drugs, colchicine, and NLRP3 inhibitor 16673-34-0 all showed a reduction in pericardial effusion or pericardial thickness. The application of anakinra and IL-1 trap also received a relief of pericarditis syndromes and the diminution of inflammasome formation in model mice. Combined with the positive therapeutic impact of colchicine and anakinra in pericarditis treatment, inhibiting NLRP3 and IL-1 pathway tends to be a promising therapeutic target for acute and recurrent pericarditis.

### Hypertension

Systemic arterial hypertension, abbreviated as hypertension, poses to be a common risk for cardio- cerebrovascular diseases and chronic kidney diseases. It is characterized as persistently high systolic and/or diastolic blood pressure in the systemic arteries (83). The pathogenesis of hypertension is diverse in different population or age stages, but inflammation has been considered as a trigger of hypertension since the bidirectional association between atherosclerosis and hypertension (84). Prior observation discovered that serum IL-1β level was elevated in patients with high blood pressure (85). This aroused the exploration of potential role of NLRP3 inflammasome in hypertension from diverse causes.

The first study investigating the correlation between NLRP3 and hypertension was in the exploration of relieving high blood pressure symptom in preeclampsia (86). In previous study, sterile inflammation was supposed to involve the pathogenesis, but whether NLRP3 inflammasome participates in the process remained undefined. Koumei Shirasuna et al. induced preeclampsia hypertension model by injecting angiotensin II in pregnant NLRP3-/- and ASC-/- mice (87). The hypertension was prevented in NLRP3-/- mice, while no significant effect of lowering blood pressure was obtained in ASC-/- mice. This result indicated that NLRP3 promotes the development of hypertension *via* an inflammasome-independent pathway. Moreover, the level of IL-6, instead of IL-1β, decreased in NLRP3-/- mice, which means that NLRP3 is widely involved in diverse inflammatory responses, and anti-NLRP3 treatment would receive further benefits of relieving auto-inflammation in hypertension pathogenesis (87).

High-salt exposure has long been viewed as an independent risk factor of hypertension. Although the specific mechanism of salt-sensitive hypertension is still under discussion, renal inflammation and oxidative stress are major factors that lead to the high blood pressure (88). As an inflammation pathway tightly associated with oxidative stress, NLRP3, and related molecules up- and down-stream involve in the development of salt-sensitive hypertension. Paraventricular nucleus is the integrating hub of endocrine system, maintaining the dynamic balance of cardiovascular system. Inhibiting NF-κB effectively alleviated the production of pro-inflammatory cytokines and oxidative stress in the paraventricular nucleus, and thus constrained salt-sensitive hypertension in rat models (9). In addition to inhibiting NF-κB, direct inhibiting IL-1β, the central pro-inflammatory cytokine in NLRP3 pathway, also obtained the results of attenuating hypertension by the activation of renin-angiotensin system and the generation of ROS in paraventricular nucleus (89).

Hypertension is not only a result of cardiovascular disorder but also a leading cause of several cardiac damages. Cardiac remodeling is a common complication of hypertension, in which renin-angiotensin system plays a central role (90). Angiotensin II (Ang II) is a vasoconstrictive peptide that promotes the activation of renin-angiotensin system. A previous study demonstrated that Ang II infusion advocated the activation of NLRP3 inflammasome and the secretion of pro-inflammatory cytokines, which mediated the inflammation process in cardiac fibrosis (91). The application of EMD638683, a selective inhibitor suppressing the activation of NLRP3 inflammasome, effectively reduced cardiac fibrosis in Ang II- induced hypertension mice model. However, the blood pressure seemed not affected in experiment. Moreover, vascular remodeling and vascular endothelial cell dysfunction are also pathogenesis resulting from hypertension, which can aggravate the development of hypertension in turn, leading to a vicious circle of elevating blood pressure pathologically. Blocking NLRP3 with sinapine thiocyanate or knocking down upstream receptor TLR4 posed a relief of endothelial dysfunction or vascular smooth muscle cell proliferation respectively (92, 93).

In aggregate, NLRP3 and its inflammasome greatly contribute to the development and further damages of hypertension. NLRP3 seems to be a novel target of hypertension treatment. However, a matured protocol of therapy is required for clinical applications.

### Abdominal aortic aneurysm

Abdominal aortic aneurysm (AAA) is a severe vascular disease characterized by the progressive pathological dilatation of the abdominal aortic wall. It is mostly asymptomatic but can be life-threatening in the case of rupture. Pathophysiology and histopathology research discovered that the development of AAA was always associated with the infiltration of inflammatory cells, loss of vascular smooth muscle cells (VSMCs), and failure of the structural proteins of the aorta (94). This indicated the involvement of the inflammatory response in the process of AAA (95). Pyroptosis is proved to be highly involved the damage of VSMCs and vascular endothelium. As the upstream trigger of pyroptosis, the NLRP3 inflammasome is a remarkable contributor to the development of AAA.

Many experiments revealed the detailed mechanism at the molecular, cellular, and organismic levels (the latter in murine models). Wu et al. discovered that the NLRP3–caspase-1 inflammasome degrades contractile proteins in human thoracic aortic SMCs under the stress created by palmitic acid (PA) (0.3 mM). Later, immunoprecipitation experiments indicated that caspase-1 bound to tropomyosin and myosin heavy chain in VSMCs and recombinant caspase-1 and directly cleaved the heavy chain. This resulted in the decrease of the contractile capability of VSMCs in a collagen matrix. In contrast, the contractile function partially improved after treatment with a caspase-1 inhibitor or knockdown of the NLRP3–caspase- 1 inflammasome cascade with siRNA (96). This indicated the NLRP3 inflammasome has a mediating function in degrading contractile proteins in VSMCs.

In 2019, Wortmann et al. applied autologous necrotic cell debris as DAMPs to stimulate human VSMCs from AAA patients (AAA-SMCs), found that NLRP3 and IL-1β mRNA and protein expression levels were higher than control cells, and the AAA-SMCs shifted to an inflammatory phenotype (97). This verified that NLRP3-mediated inflammation was involved in the dysfunction and pyroptosis of VSMCs, contributing to the development of AAA.

In experiments with mice, the role of the inflammasome in the pathogenesis of AAA has been fully exposed. In 2016, Wakita et al. elucidated the role of IL-1 signaling in AAA in a mouse model of Kawasaki disease. Model mice developed severe AAA in the control group. In comparison, IL-1α-/-, IL-1β-/-, Casp1-/-, and Nlrp3-/- mice were well protected against abdominal aorta dilatation under the same stimuli. Injection with neutralizing antibodies also obtained protective results similar to those observed in gene-deficient mice (98). In another experiment, Ren et al. applied MCC950 as a selective NLRP3 inhibitor and explored the difference in AAA formation in wild-type mice fed with a high-fat and a high-cholesterol diet. 98% of the mice developed aortic dilatation in the control group while 87% of mice developed aortic dilatation after intervention (*P* = 0.03). Matrix metallopeptidase 9 (MMP-9) is an apoptosis-associated molecule and is activated by the NLRP3 inflammasome. Results showed that the levels of IL-1β, caspase-1, and activated MMP-9 were significantly lower in mice treated with MCC950 compared to the control group (99).

### Myocardial infarction

Myocardial infarction (MI), also known as a “heart attack,” is a secondary symptom of inadequate blood supply to the myocardium, leading to heart dysfunction, hemodynamic deterioration, and even sudden death (100). Due to the limited regenerative ability of myocardium, reperfusion therapy is the best treatment for myocardial ischemia symptoms. However, reperfusion is usually coupled with severe inflammation, resulting in ischemia/reperfusion (I/R) injury and causing even greater damage to heart tissue. NLRP3 inflammasome is a significant contributor to infarction size during the I/R process.

During the examination of cardiac tissues from patients who died from MI, infiltration of inflammatory cells and high expression of ASC were observed (101), suggesting that the NLRP3 inflammasome might be involved in I/R injury. In 2011, Kawaguchi et al. were the first to reveal the role of the inflammasome in myocardial I/R injury (101). They applied bone marrow transplantation in wild-type mice to create ASC- deficient mice, occluded the left anterior descending artery for 30 min, and reperfused for 48 h to establish an I/R model. The results revealed that ASC-/- mice had smaller infarcted areas and exhibited decreased ventricular remodeling (101). Sandager et al.'s study concluded with a similar viewpoint. The mRNA expression of NLRP3, IL-1β, and IL-18 mRNA was upregulated in mice after MI surgery (102). In the *ex vivo* Langendorff model, NLRP3 inflammasome was upgraded in myocardial fibroblasts, NLRP3-/- mice had smaller areas of infarction. However, it remained controversial that the expected outcome was not observed in ASC-/- mice in the same experiment (102). A reasonable explanation is that ASC may also participate in the assembly of several other inflammasomes that play a protective role. This also indicated the inflammasome-independent effects of NLRP3 itself (102). An *in vitro* study pointed out that the inflammatory response could be triggered in cardiac fibroblasts (instead of cardiomyocytes) by PAMPs like LPS, cellular ROS generation, and potassium efflux (101). However, another experiment discovered that cardiomyocytes also contained the structural components of the NLRP3 inflammasome in MI mice (103). Nonetheless, both of them further proved the widespread existence of NLRP3 in cardiovascular and inflammatory cells.

### Stroke

Stroke is a common cardio-cerebrovascular disease, affecting one of four people all around the world. Although great strides in progress were obtained in past years, it remained the second leading cause of death and third leading cause of disability in adults. The motivation of stroke can be attributed to ischemia and rupture of cerebral arteries, and 80–90% of stroke presentations are ischemic due to arterial occlusion. The chronic damages of stroke usually come from the ischemia/reperfusion injury following brain tissue ischemia. Highly involved in the development of atherosclerosis and ischemia/reperfusion injury, NLRP3 inflammasome has gained much attention in study of stroke pathogenesis.

Early evidence showed that activators of NLRP3 played a remarkable role in inflammation process of ischemic stroke, and several studies have explored the detailed mechanism of inflammatory response in this process (104–106). Yang et al. in 2014 demonstrated that NLRP3 was expressed in microglia and endothelial cells of and upregulated in ischemic brain (107). NLRP3-deficient mice received reducing infarcts and less damage on brain-blood-barrier after ischemic stroke *via* the examination of magnetic resonance imaging. Pro-inflammatory cytokine IL-1β levels up and has been proved to aggravate stroke pathogenesis. Inhibiting IL-1β signaling *in vivo* received an improved result after stroke (108). More studies focusing on small molecular inhibitors of NLRP3 inflammasome or its downstream products could also draw similar conclusions that inhibiting NLRP3 inflammation contributed to the relief of brain injury and vicious neuroinflammation after stroke (109–111).

In addition to the basic mechanism research, the results in clinical inspection of peripheral circulating plasma from patients who suffered from stroke also emphasized the significance of NLRP3 in the prognosis of ischemic stroke. Li et al.'s study in 2020 demonstrated that the NLRP3 mRNA level had a negative correlation to the outcome of stroke patients, and the 2high NLRP3 mRNA level was an independent risk factor for poor prognosis (112). This finding strongly supported the hypothesis that NLRP3 involved in development and pathological injuries of ischemic stroke *via* human experiments. Targeting NLRP3 might be a novel choice for the prevention and recovery of stroke.

### Thrombosis

Thrombosis is a multifactorial disease resulting in the condition of thrombus formation in blood vessels. Virchow Triad has described the interaction of three categories of factors that contribute to thrombosis: flow, vessels, and blood (113). The formation is a process of dynamic balance, which could falls into accumulation when this dynamic balance is disturbed. Previous studies have shown that there is a strong dichotomy between thrombosis and a variety of inflammatory diseases, in which NLRP3 inflammasome have a prominent prothrombotic phenotype in the disease development, contributing to the positive loop and mutual amplification of coagulation process (114–116). *Gupta* et al. has demonstrated that stress conditions such as hypoxia contributes to the accumulation of thrombus induced by activation of NLRP3 inflammasome (117). Although the involvement of NLRP3 in thrombosis has been widely recognized, the exact inflammatory pathway varies in different situations.

Normal vascular endothelium maintains the integrity and normal morphology of vessels and release substances to generate a local fibrinolytic state. However, certain stimuli could induce inflammatory response in endothelial cells, including the activation of NLRP3 inflammasome. It is proved to be associated with endothelial damage and release of pro-inflammatory substance in several studies. The activation of NLRP3 was observed in endothelial cell inflammatory response to adipokine visfatin and subsequent production IL-1β was detected under the pro-inflammatory stimuli of heme (118, 119). Moreover, microparticles from monocyte has been reported to activated endothelial cells via NLRP3 inflammasome, which activates the NF-κB pathway and leads to the expression of intercellular adhesion molecule-1, vascular cell adhesion molecule-1, and E-selectin (120). Together with the function of IL-1β in elevating membrane surface adhesion molecules, they promote the adhesion deposition of immune cells and platelets with eventual thrombosis.

Platelets play a central role in thrombosis through a series of complex biological pathways. Among them, NLRP3 inflammasome pathway significantly mediates the activation of platelet and subsequent formation of thrombus. In the *in vivo* experiment, NLRP3 inflammasome activated by Bruton tyrosine kinase in platelet, which finally led to the thrombosis (121). The research in arterial thrombosis also obtained similar results. A remarkable reduction in αIIbβ3 signaling transduction was observed together with the the release of IL-1β in NLRP3 deficient platelets, suggesting NLRP3 inflammasome contribute to the hemostasis and arterial thrombosis with the mediation of IL-1β release and αIIbβ3 signaling (122).

In addition to the exploration of specific mechanisms, promising results have been reported from studies applying miRNAs to target NLRP3 activation and thus inhibit thrombosis (123–125). These results emphasized the paramount role of NLRP3 in thrombosis, and provided with a novel perspective of treatment.

### Dilated cardiomyopathy

In epidemiology research, dilated cardiomyopathy (DCM) has been one of the most common causes of heart failure (HF) and the most common indication for heart transplantation worldwide (126). It is characterized by left ventricular dilatation and contractile dysfunction without abnormal loading conditions and severe coronary artery disease (127). The causes of DCM are complex; they are mainly divided into the genetic kind and the acquired kind, including myocarditis, exposure to alcohol, drugs, toxins, and metabolic and endocrine disturbances (126). Generally, it is mainly attributed to cardiomyocyte pyroptosis, in which NLRP3 plays an irreplaceable role.

One experiment in DCM heart supports the significant role of NLRP3 inflammasome-mediated caspase-1-dependent cardiomyocyte pyroptosis in DCM (128). Nine human heart tissue samples were examined and triple-immunostaining was performed for active caspase-1, TUNEL, and α-actinin. Pyroptotic cell death, more than apoptotic cell death, was observed in all DCM patients but absent in control hearts (128). The plasma IL-1β and IL-18 levels of DCM patients were also examined, in which both plasma levels and expression or phosphorylation of NF-κB were significantly higher compared to the healthy controls (128), indicating the significance of the NLRP3 inflammasome in the process of DCM.

In addition to the inflammatory reaction, NLRP3 also promotes the fibrosis in development of DCM. One experiment revealed that the knockdown of NLRP3 with siRNA could restore the expression of collagen I and III in the myocardium of a DCM rat model (129). Other studies also revealed that NLRP3 could promote fibrosis and pro-fibrosis through several pathways such as the Smad (R-Smad) pathway and cAMP signaling in fibroblasts (130, 131). Pharmacologic or genetic inhibition of NLRP3 was confirmed to improve fibrosis in myocardium (129). The fibrosis process reduces the contracting ability of cardiomyocytes, leading to myocardial diastolic and systolic contractile dysfunction.

### Diabetic cardiomyopathy

CVDs increase the mortality of patients with diabetes mellitus (DM) (132); approximately 50% death of DM patients attribute to various CVDs. Besides the widely known coronary artery disease, cardiomyopathy, which eventually leads to HF, also contributes significantly to the death of DM patients. Diabetic cardiomyopathy (DCM) is characterized by diastolic and systolic contractile dysfunction, interstitial fibrosis, and an increase of ventricular mass (mainly the left ventricle) (133). Pyroptois is the major contributor of the pathogenesis of DCM (129), in which the NLRP3 inflammasome plays a critical role as mentioned above (134). Although hyperglycemia has been proved to be one of the triggers of NLRP3 inflammasome activation (135), the specific details of the relationship between hyperglycemia and the inflammatory response have not been thoroughly explored.

DM is classified into type 1 diabetes (T1D) and type 2 diabetes (T2D). Both T1D and T2D result in hyperglycemia while T2D can lead to hyperlipidemia additionally. Glucotoxicity and lipotoxicity are notable signals in the process of NLRP3 inflammation in DCM, involving both priming and activation stages. Several studies demonstrated that high glucose and lipid levels in blood could promote the overexpression of ROS (130), which leads to the activation of the NF-κB pathway and promotes the transcription of NLRP3 and pro-IL-1β and pro-IL-18 (136). Thioredoxin interacting/inhibiting protein (TXNIP) is the second signal in the activation of NLRP3, binding to it directly to modulate its oligomerization (137). ROS generation resulting from hyperglycemia could upregulate the expression of TXNIP, facilitating the activation through another pathway. Besides the classic pathway of inflammation, hyperglycemia and hyperlipidemia (including fatty acids [FAs]) also contribute to the process through bypass pathways, exacerbating the mitochondrial oxidative stress and other pro-inflammatory cytokines, in turn inducing the formation of the NLRP3 inflammasome.

Besides the classic ROS-mediated activation pathway, cytosolic Ca2+ involves and effectively promotes the activation of NLRP3 inflammasome (138). Sarcoplasmic/endoplasmic reticulum calcium ATPase 2 (SERCA2a) is an important enzyme that maintains the transport between reticulum and cytoplasm. Its dysfunction leads to Ca2+ transport disorder and results in NLRP3 activation and subsequent pyroptosis. The sarcoplasmic reticulum function reduced synchronously with the SERCA2a levels in T1D rats (139). Data from T2D mice also received similar results of reduction of SERCA2a expression levels. However, increased expression levels of SERCA2a in DM cardiomyocytes effectively ameliorated contractile function in contraction experiments.

### Heart failure

Heart failure (HF) is an end-stage syndrome of almost all kinds of severe CVDs, resulting from various cardiac impairments of the heart's structure or function. HF is characterized by the inability of maintaining normal blood output levels caused by contractile or diastolic dysfunction, leading to high mortality for CVDs (140). The underlying mechanism of HF is acceptedly attributed to myocardial remodeling, in which inflammatory cytokines play an important role (141). Two physiological events have been recognized as the central step in the remodeling process: myocardial injury caused by pyroptosis and the activation of cardiac fibroblasts.

Classic triggers like ROS and oxLDL-C have already been deeply reviewed in past studies. The novel discovery of NO physiological functions provides another possible pathway of apoptosis mediated by inflammatory cytokines. Low levels of NO generated from the constitutive NO synthase play a protective role to cardiomyocytes by relaxing vessel smooth muscles or other pathways (142). But abnormally high levels of NO generated by enzymes such as inducible NO synthase (iNOS) lead to myocardial injury and mediate leukocyte-endothelial interactions (143). IL-1β is a potent inducer of iNOS while IL-18 promotes the overexpression of iNOS (144, 145). Both of their maturations require the cleave of caspase-1 on the NLRP3 inflammasome (146, 147). Excessive iNOS level leads to not only the overproduction of NO and subsequent apoptosis and remodeling, but also the synthesis of small uncharged NO- molecules. NO- molecules can morph into reactive nitrogen species (RNS), play similar role as ROS (148).

In addition to the NO-mediated cell death promoted by the IL-1 family, other inflammatory cytokines also play roles in the process of HF. Tumor necrosis factor α (TNF-α) is a significant pro-inflammatory cytokine that directly leads to cell hypertrophy and apoptosis (149, 150). In the normal myocardium, TNF-α is not observed. However, in HF animal models, TNF-α is found in cardiomyocytes, decreasing cardiac contractility by decreasing intracellular Ca2+ release (101, 151). IL-18, one of the downstream products of NLRP3 activation, contributes to the production of TNF-α. Meanwhile TNF-α can trigger the NF-κB pathway, leading to NLRP3 and IL-1 transcription in turn. This cross-interaction aggravates the inflammatory injury of myocardial tissue.

Fibrosis is the central process in ventricle remodeling. Fibroblasts make up two-thirds of cardiac tissue, playing a critical role in ventricle fibrosis (101). The lack of oxygen promotes the production of ROS and the efflux of K+ from cardiac fibroblasts, which are known to be significant triggers of the NLRP3 inflammasome (152). The NLRP3 inflammasome is the initial sensor of DAMPs after hypoxia. Via the activation of the NLRP3 inflammasome, the fibroblasts induce and enhance the inflammatory impairment after myocardial ischemic injury. In addition to the inflammatory reaction, hypoxia also leads to the fibrogenic phenotype in cardiac fibroblasts, resulting in myofibroblast trans-differentiation and an increase in collagen production (152). These ultimately lead to inocyte multiplication, myocardium fibrosis, and finally ventricle remodeling. Recent studies also discovered that NLRP3 can promote inflammasome- independent cardiac fibroblast differentiation through its structure (153). It has been found that NLRP3 regulates mtROS levels and enhances R-Smad signaling, which eventually leads to the expression of pro-fibrotic genes. This provides a novel pathway of myocardial fibrosis via NLRP3, which contributes to the remodeling and finally leads to HF.

### Calcific aortic valve disease

Calcific aortic valve disease (CAVD) is one of the most common human valve diseases, leading to aortic stenosis (AS) and subsequent adaptive responses like left ventricular hypertrophy and HF (154). To date, there is no effective pharmacological therapy for CAVD but surgical or interventional aortic valve replacement (155).

Similar to the discovery of the pathological mechanism of atherogenesis, the calcification of aortic valves was initially attributed to the degenerative osteogenic processes and passive substance deposition. However, recent studies pointed out that positive processes such as chronic inflammation, lipoprotein deposition, and osteoblastic differentiation could be the exact fundamental cause of CAVD (156, 157). Nonetheless, the mechanism underlying the inflammatory processes in CAVD remains to be integrally revealed in detail.

The human aortic valve is composed of four types of cells: valve endothelial cells (VECs), valve interstitial cells (VICs), cardiac muscle cells, and smooth muscle cells. VICs are the most common cells in valve tissue, in contrast to other mesenchymal cell types in other organs (158). They are usually divided into five subtypes based on their different functions and gene expression levels: embryonic progenitor endothelial/mesenchymal cells, quiescent VICs (qVICs), activated VICs (aVICs), progenitor VICs (pVICs), and osteoblastic VICs (obVICs) (159). These forms are not absolute and can transform from one to another. Under normal conditions, VICs usually remain in quiescence to maintain normal valve physiology. When valve injury occurs, qVICs can be activated into aVICs by the inflammatory response and cytokines (157). aVICs have a similar function to mesenchymal repair cells, differentiate into myofibroblast type cells, and express the marker alpha-smooth muscle actin (α-SMA) after stimulation (160). Cell proliferation and extracellular matrix remodeling occur after the activation of aVICs, leading to valve architectural disruption and subsequently valve cusp thickening or fibrosis. This worsens the injury of qVICs and VECs in turn, creating a vicious circle and impeding restoration to physiological conditions. After accumulation of impairments, osteogenic differentiation, activation of obVICs, and calcification of the aortic valve would be observed.

Initially, inflammation did not attract much attention. However, an abnormal phenomenon provided a novel mechanism of CAVD: inflammatory cells, such as macrophages, T lymphocytes, and mast cells, were found in calcific valves, which would not represent in normal ones (161, 162). This indicates that inflammation may be a critical factor causing CAVD. Although the exact mechanism by which the NLRP3 inflammation contributes to the development of CAVD has not been thoroughly explored yet, one recent experiment suggested the involvement of NLRP3 (163). Caffeic acid phenethyl ester (CAPE) is a natural polyphenolic compound found in conifer trees' bark and honeybee hives (164) that suppressed the formation of calcified nodules. Li and Liu et al. furthermore found that the phosphorylation of NF-κB was inhibited by the addition of CAPE (163). They cultured human aortic valvular interstitial cells to evaluate the effect of CAPE on osteogenesis and the activation of the NF-κB pathway. It was observed that CAPE inhibited the phosphorylation of AKT, ERK1/2, and IκBα, blocking the activation of the NF-κB pathway, inhibiting the transcription of NLRP3, and potently suppressing the subsequent inflammation. Additionally, IL-18, one major product of NRLP3 inflammasome, has also been proved to be a potential mediator of aortic valve calcification (165). These indicated that NLRP3 inflammation might play a role in the development of CAVD; however, the entire details of the signal pathway require further investigation.

There have remained no available drug treatment options for CAVD to date. However, research into the underlying pathogenesis of CAVD have provided us with new therapeutic perspectives. Other than inhibiting NLRP3 with CAPE, CY-09 also posed a protective effect on calcified aortic valve stenosis in one recent study (166). Moreover, in research on bioprosthetic heart valve, the major valve substitutes for valve replacement surgery, outcomes from 2518 patients undergoing TAVR demonstrated that statin treatment resulting significantly less BHV calcification (167). Given the anti-inflammatory effect of statin, immunosuppressive therapies, including directly NLRP3 inflammasome or its downstream products, rise as a promising therapeutic strategy for valve calification.

## Possible therapies associated with the NLRP3 inflammasome

Since the role of inflammation in CVDs has gradually been confirmed (168), many experiments have been carried out to explore possible treatment strategies targeting the inflammatory reaction. Considering that NLRP3 is a novel mediator of the innate immune response, inhibition of the NLRP3 inflammasome or its upstream regulators and downstream effectors has attracted much attention. Several inhibitors targeting NLRP3 inflammasome and IL-1β received effects of relief in clinical trials in recent years (Table 1).

**Table 1:** Overview of completed clinical trials and major outcomes.

**Clinical trials**	**Intervention**	**Targeted diseases**	**Number of patients**	**Major outcomes**	**Major serious adverse outcomes**	**Sponsor**	**Results first posted year**	**PMID or NCT number**
Interleukin-1 (IL-1) blockade in acute myocardial infarction (VCU-ART3)	Anakinra	Acute myocardial infarction	99	Acute phase response (CRP Levels)	Infection	Virginia Commonwealth University	2019	NCT01950299
Anakinra to Prevent Adverse post-infarction Remodeling (2)	Anakinra	Acute myocardial infarction, heart failure	30	Change in left ventricular end-systolic volume indices	Serious infection	Virginia Commonwealth University	2016	NCT01175018
Treatment of Acute Pericarditis With Anakinra	Anakinra	Acute Pericarditis	5	Pain Score Change	Thrombophlebitis	Virginia Commonwealth University	2021	NCT03224585
Interleukin-1 blockade in HF with preserved EF	Anakinra	Heart failure	31	Change in ventilatory efficiency	Acute decompensated heart failure	Virginia Commonwealth University	2018	NCT02173548
Interleukin-1 blockade in recently decompensated heart failure	Anakinra	Heart Failure	60	Interval changes in peak oxygen consumption (vo2)	Serious infection, acute kidney injury	Virginia Commonwealth University	2017	NCT01936909
Interleukin (IL)-1 blockade in acute heart failure (Anakinra ADHF)	Anakinra	Heart failure	30	Plasma C reactive protein levels	Serious infection/sepsis	Virginia Commonwealth University	2016	NCT01936844
Anakinra to prevent post-infarction remodeling (VCU-ART)	Anakinra	ST segment elevation acute myocardial infarction	10	Change in end-systolic volume indices	-	Virginia Commonwealth University	2010	NCT00789724
ACZ885 for the treatment of abdominal aortic aneurysm	Canakinumab	Abdominal aortic aneurysm	65	Change from baseline in abdominal aortic aneurysm (AAA) size per year	Hip fracture	Novartis Pharmaceuticals	2016	NCT02007252
Safety and effectiveness on vascular structure and function of ACZ885 in atherosclerosis and either T2DM or IGT patients	Canakinumab	Atherosclerosis	189	Change in aortic distensibility and plaque burden	Infections, nervous system disorders	Novartis Pharmaceuticals	2015	NCT00995930
Canakinumab anti-inflflammatory thrombosis outcome study	Canakinumab	Cardiovascular events	10,061	non-fatal MI, non-fatal stroke, cardiac death	infection/sepsis	Novartis Pharmaceuticals	2,017	28845751
Colchicine cardiovascular outcomes trial (COLCOT)	Colchicine	Recent acute myocardial infarction	4,745	Ischemic cardiovascular events	Pneumonia	Montreal Heart Institute	2020	NCT02551094
Low-dose colchicine	Colchicine	Stable coronary artery disease	532	Cardiovascular events	-	Heart Research Institute of Western Australia	2013	23265346
Low-dose colchicine 2	Colchicine	Chronic, stable coronary artery disease	5,522	Cardiovascular events	non–CVDrelated deaths	Heart Research Institute of Western Australia	2020	32865380
Colchicine for left ventricular infarct size treatment in acute myocardial infarction	Colchicine	First stable ST segment elevation acute myocardial infarction	192	Infarct size, left ventricular remodeling, left ventricular end-diastolic volume	Left ventricular thrombus, gastrointestinal adverse events	French Ministry of Health	2021	34420373
Study to assess the efficacy and safety of rilonacept treatment in participants with recurrent pericarditis	Rilonacept	Recurrent pericarditis	86	Time to pericarditis recurrence	Cardiac flutter, squamous cell carcinoma	Kiniksa Pharmaceuticals (UK), Ltd.	2021	NCT03737110
Rilonacept to improve artery function in patients with atherosclerosis	Rilonacept	Atherosclerosis	10	C-reactive protein levels	Upper respiratory infection	National Heart, Lung, and Blood Institute (NHLBI)	2010	NCT00417417

### Anakinra

Anakinra is a selective antagonist of IL-1 receptor prepared by gene recombination technology, blocking the activity of both IL-1α and IL-1β. Existing clinical trials of Anakinra in CVDs are most focused on pericarditis, heart failure, and myocardial infarction.

A small-scale trial published in 2021 investigated the acute efficacy of anakinra in patients with acute pericarditis (169). Five volunteers were recruited, and pain scores were evaluated compared to the baseline measure. The results showed that Anakinra significantly reduced pain scores from 6.0 to 4.0 at 6 h (*P* = 0.012) and to 2.0 at 24 h (*P* = 0.0025) in patients administered with 100 mg Anakinra. The trial was terminated due to the dramatic benefits in the first 24 h, providing a bright further of anakinra in treatment of pericarditis.

Three trials of anakinra concerning the treatment of heart failure comprehensively evaluated the change in ventilatory efficiency, interval changes in peak oxygen consumption (VO2), and plasma C reactive protein levels (170, 171). The results posed that anakinra contributed to the improvement of systemic inflammatory, and NT-proBNP also posed a favorable trend after anakinra. Despite VO2 seemed not affected in trial, the ejection fraction was preserved or improved in volunteers receiving anakinra.

Additionally, the potential of anakinra application in treatment of MI is also evaluated. One group performed three phases of trials (VCU-ART) and thoroughly evaluated the safety and efficacy of anakinra in acute myocardial infarction treatment (171–173). The trials enrolled a total of 139 patients and results demonstrated that anakinra significantly reduced the systemic inflammatory response after MI. Although the efficacy of anakinra seemed to be neutral in improvement of recurrent ischemic events and left ventricular ejection fraction, the treatment group had less risk of heart failure after acute MI.

However, although Anakinra has a promising future in anti-inflammatory therapy and has been approved for the treatment of several immune-related diseases, none of Anakinra treatments have been approved for treatments of CVDs such as pericarditis. Further large-scale clinical trials are required to provide solid support for safe application of Anakinra in CVDs treatments.

### Colchicine

Colchicine is a widely applied anti-inflammatory drug in treatment of pericarditis and gout. It inhibits the formation of microtube and subcellular transport of ASC and NLRP3, and further blocks the assembly of inflammasome. Although colchicine is recognized as the first-line treatment for pericarditis, the feasibility of its application in treatment of other CVDs is still under exploration. Since colchicine is a safe and inexpensive antiinflammatory drug, several large-scale clinical trials have been conducted in attempt of relieving different kinds of CVDs.

The Low Dose Colchicine (LoDoCo) study is the first large trial with cardiovascular endpoints, randomizing 532 patients with stable coronary artery disease into colchicine or placebo groups (174).

All volunteers were followed up for the median of 3 years, and the group of colchicine treatment posed a significantly lower risk of cardiovascular events. The result of LoDoCo2 trial also validated the similar conclusion, which randomized 5,522 volunteers and observed a 31% decrease of primary composite endpoints, such as cardiovascular death and ischemia-driven coronary revascularization, after colchicine treatment (175).

The potential of colchicine in MI treatment also received much attention in recent trials. Colchicine Cardiovascular Outcomes Trial (COLCOT) unblinded in 2020 revealed a decrease of incidence in coronary and cerebral atherothrombotic events in colchicine treating group compared to placebo (176). A later relatively small study was conducted focusing on first stable ST-segment elevation acute MI. However, the result of trial demonstrated no difference in infarct size, left ventricular remodeling, and left ventricular end-diastolic volume in 5 days and 3 months after MI (177).

Above evidence indicated that colchicine might be a promising treatment of various CVDs, however, more studies are required for further confirmation of colchicine treatment effect in the specific CVD. Several ongoing trials also focus on the prevention of CVDs. Their results may bring about a novel perspective of colchicine application in CVDs.

### Canakinumab

Canakinumab is a monoclonal antibody that directly neutralizes IL-1β. Among the clinical trials of canakinumab, the Canakinumab Anti-Inflammatory Thrombosis Outcomes Study (CANTOS) is the one most discussed (178).

The trial began in late 2011, randomizing 17,200 adult men and women who suffered from acute MI for more than 29 days before the study, and finally 10,061 volunteers were enrolled for further investigation. The selected volunteers had hsCRP levels over 2.0 mg/L despite the stable application of standard secondary prevention treatments. To evaluate the optimal amount of canakinumab, the CANTOS trial set three doses: 50, 150, and 300 mg subcutaneous injection every 3 months. The first endpoint was set as non-fatal MI, non-fatal stroke, or cardiovascular death.

The results revealed in 2017 showed that the patients treated with 150 mg canakinumab every 3 months had a 15% reduction of recurrence compared to the placebo group. Although this result is not significant, canakinumab tops the secondary prevention therapies, providing a feasible supplement to standard statin therapy. Besides, CANTOS is the first randomized controlled, large-scale, double-blinded, international clinical trial that systematically targets one specific inflammatory cytokine in the process of CVDs. The CANTOS results strongly emphasized the significant role of IL-1β in the development of CVDs, confirming the possibility of improving the outcome of post-MI patients via targeting IL-1β function or production.

Besides the predictable results in alleviating CVDs, CANTOS provided another unexpected finding: the all-cause mortality rate was unaffected in the CANTOS trial, despite the weakened innate immune system diminished by blocking IL-1β. This unforeseen result came from the reduction of cancer mortality. The mortality of lung cancer was significantly reduced among the CANTOS population (179). One of the reasonable explanations is that IL-1β participates in the construction of an inflammatory tumor microenvironment, promoting carcinogenesis in several cancers (179). The reduction of lung cancer mortality in the CANTOS trial indicated that IL-1β-targeting therapy might also benefit patients with certain cancers.

Besides CANTOS, another trial enrolling 189 volunteers also explored the feasibility of blocking IL-1β in treatment of atherosclerosis. Although no statistically significant change in mean carotid wall area and aortic distensibility were observed in the trial, canakinumab effectively reduced markers of inflammation such as hsCRP and IL-6. Lipoprotein levels were also reduced compared to placebo group. However, the levels of total cholesterol and triglycerides were upregulated by canakinumab.

In addition to MI and atherosclerosis, the application of canakinumab in alleviating abdominal aortic aneurysm has also been explored. Volunteers treated with canakinumab posed a non-significant reduction in aortic diameters. However, due to the lack of efficacy and futility, this trial was terminated in third interim analysis. The validity of results and effectiveness of canakinumab treatment in abdominal aortic aneurysm is still under skepticism.

After all, canakinumab is a promising IL-1 pathway inhibitor for the treatment of CVDs, including MI, atherosclerosis, and abdominal aortic aneurysm. However, more large-scale and comprehensively examined clinical trials are required to provide evidence supporting the application of canakinumab in CVDs.

### Rilonacept

Rilonacept is a dimeric fusion protein, composing the extracellular portion of the IL-1 receptor and the Fc portion of IgG1. It acts as a soluble decoy receptor, preventing interaction of IL-1β with cell surface receptors. Although the study on applying Rilonacept in CVDs treatment has gained attention early, the results of clinical trial are indeed limited.

One small-scale trial in 2010 enrolling 10 volunteers investigated the treatment effect of Rilonacept in atherosclerosis (180). A reduction of CRP was observed in treatment group compared to placebo one. However, the difference posed no statistical significance between groups. A recent trial started in 2019 focusing on recurrent pericarditis demonstrated that Rilonacept effectively alleviated the pain and inflammation of pericarditis (181). The reliance on corticosteroids was successfully tapered or lifted and health-related quality of life improved while bringing about no increasing safety concerns. In 2021, Rilonacept treatment was approved by the U.S. FDA as the first therapeutic agent for preventing recurrent pericarditis in patients over 12-year-old.

One of the major limitations of the development in NLRP3-pathway-targeting treatment is that there remains no specific NLRP3 inhibitor being approved by FDA for CVD therapies. Nonetheless, several clinical trials have been conducted to identify the efficacy of NLRP3 inhibitors in CVDs, including dapansutrile and tranilast. The revealed results exposed a promising efficacy of NLRP3 inhibitor treatment in the relief of CVDs, and the treatment was safe and well-tolerated in volunteers (182). People will benefit if feasible and specific therapies are designed and put into practice.

## Conclusion

NLRP3 is a significant PRR in the innate immune system, which can be activated by various PAMPs and DAMPs. The formation of the NLRP3 inflammasome induces the production, cleavage, and release of IL-1β and IL-18, leading to low-grade chronic inflammatory reactions. Recent studies revealed that inflammation contributes to the development of multiple CVDs. The mechanisms have been deeply investigated. They could be generally summarized as follows: multiple triggers activate the transcription process via the NF-κB pathway or the assembly of the NLRP3 inflammasome, which induces the production, maturation, and release of IL-1β; in turn, IL-1β promotes the transcription *via*/of IL-1R. This vicious circle results in damage of vascular endothelial cells, smooth muscle cells, and cardiomyocytes. In related experiments, blocking the NF-κB pathway, binding IL-1β, and silencing NLRP3 gave positive results in the prevention and control of CVDs. Moreover, patients with certain cancers, especially lung cancer, can also benefit from IL-1β-targeting anti-inflammatory therapy. Among many inhibiting schemes, directly inhibiting the NLRP3 inflammasome seems to have minimal side effects and is expected to achieve maximum preventive effects. Some natural products also show anti-inflammatory effects, which contribute to the treatment of related diseases. Although specific feasible therapy is still underexplored, targeting the NLRP3 inflammasome provides a novel, promising way of treatment and secondary prevention of CVDs and some immune-related diseases, including certain severe cancers. Direct and specific inhibitors of NLRP3 and well-designed therapy plans will boost the clinical treatment of inflammasome-related disorders and their related diseases.

## Author contributions

YZ and LX was in charge of searching all the relative papers and writing this manuscript. FL and ND gave their valuable and professional suggestions, guide in organizing, and drafting this manuscript. All authors contributed to the article and approved the submitted version.

## Funding

This study was supported by the National Natural Science Foundation of China (81974034).

## Conflict of interest

The authors declare that the research was conducted in the absence of any commercial or financial relationships that could be construed as a potential conflict of interest.

## Publisher's note

All claims expressed in this article are solely those of the authors and do not necessarily represent those of their affiliated organizations, or those of the publisher, the editors and the reviewers. Any product that may be evaluated in this article, or claim that may be made by its manufacturer, is not guaranteed or endorsed by the publisher.
